# Fetal Arrhythmia Leading to a Diagnosis of Congenital Long QT Syndrome Type II

**DOI:** 10.1016/j.jaccas.2023.102218

**Published:** 2024-01-11

**Authors:** Aidan Milner, Lindsey R. Mitrani, Lauren Ferrara, Anuradha Lala, Leslee J. Shaw, Barry Love

**Affiliations:** a Zena and Michael A. Wiener Cardiovascular Institute, Icahn School of Medicine at Mount Sinai, New York, New York, USA; b Division of Maternal and Fetal Medicine, Department of Obstetrics, Gynecology, and Reproductive Science, Icahn School of Medicine at Mount Sinai, New York, New York, USA

**Keywords:** congenital long QT syndrome, fetal ventricular tachycardia

## Abstract

We describe the case of an asymptomatic young pregnant woman with a diagnosis of congenital long QT syndrome type II in the context of in utero fetal 2:1 heart block and ventricular tachycardia. The presentation, clinical considerations, and management of the mother and baby in the antepartum and postpartum periods are discussed.

## Graphical abstract

**Figure:**
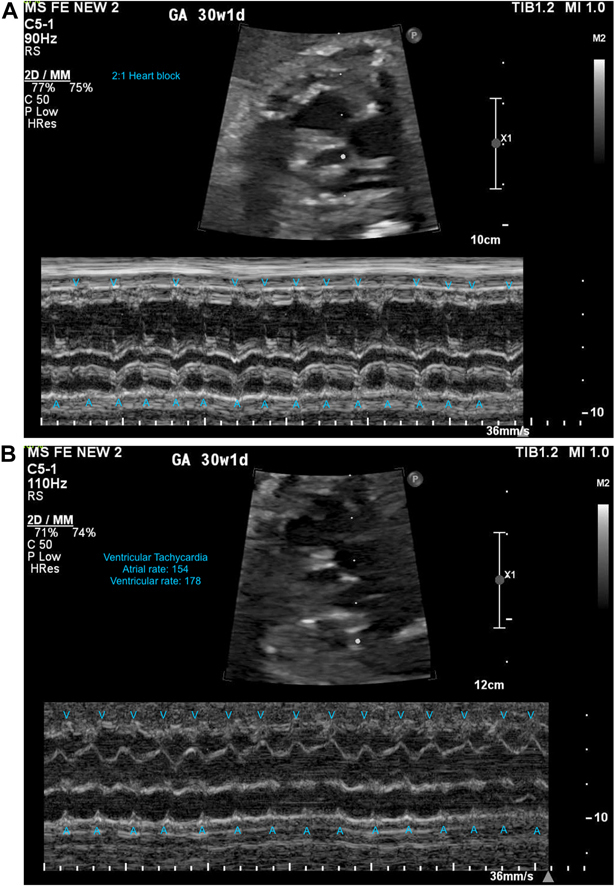

## History of Presentation

**Figure 1:**
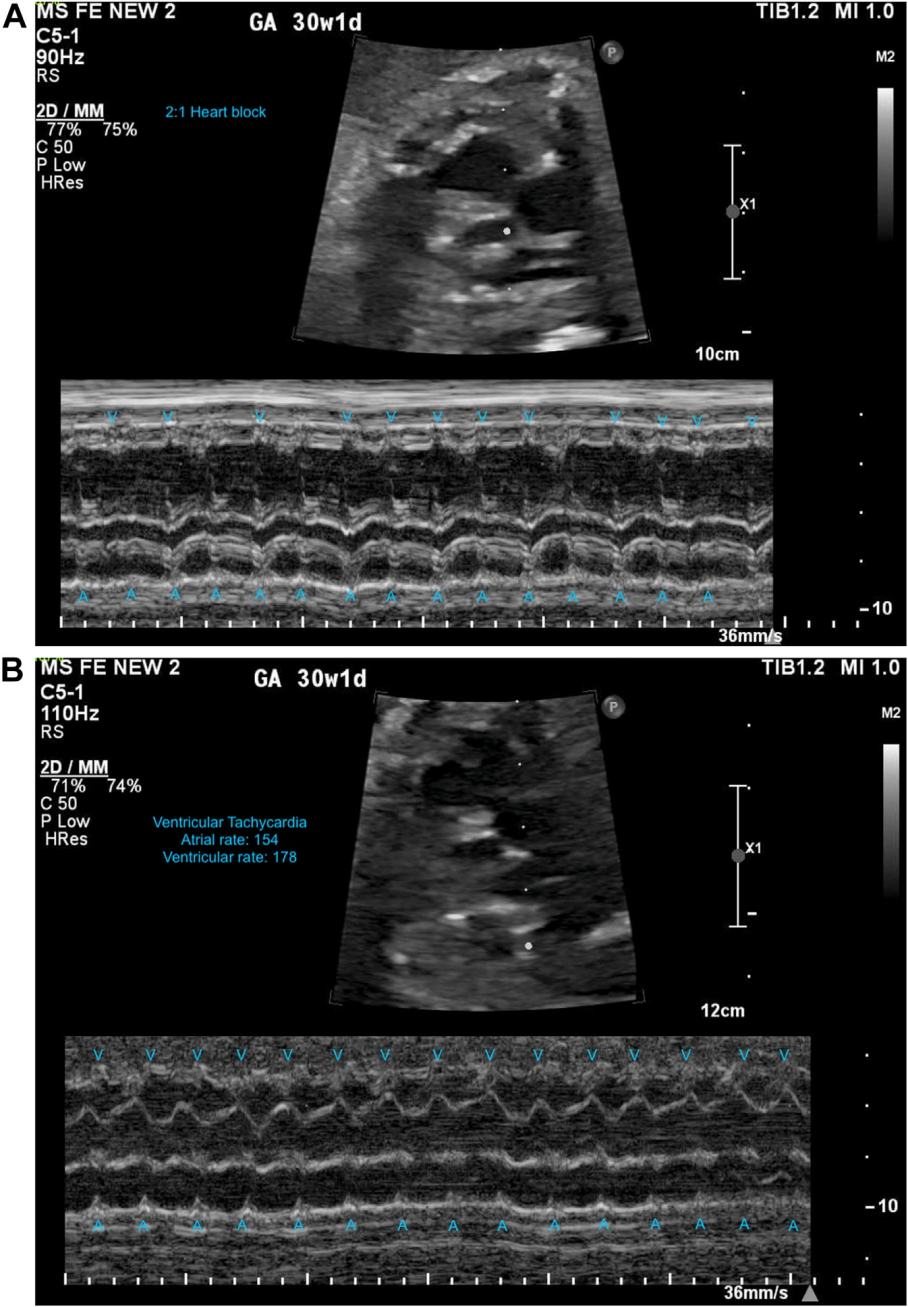
Fetal Echocardiogram at 30 Weeks 1 Day M-mode echocardiogram annotated with ventricular (V) and atrial (A) contractions. (A) Episodes of 2:1 atrioventricular block. (B) Ventricular tachycardia. GA = gestational age.

A 39-year-old G4P2010 woman presented for a routine prenatal visit at 28 weeks’ gestation where the fetal heart rate was bradycardic at 70 beats/min and a fetal echocardiogram revealed a structurally normal heart with 2:1 alternating with 1:1 atrioventricular (AV) conduction (at 148 beats/min). She was empirically treated with dexamethasone and intravenous immunoglobulin (IVIG) while awaiting laboratory evaluation for maternal autoantibodies. At 30 weeks, the fetus had similar patterns in AV conduction (Figure 1A) with new episodes of ventricular tachycardia (VT) (Figure 1B). Additional findings included a mild pericardial effusion, without evidence of hydrops fetalis. The mother was asymptomatic, with unremarkable vital signs and physical examination, and was subsequently admitted for management.

**Learning Objectives**

• To be able to create a differential diagnosis for fetal heart block and VT.
• To understand the evaluation and management of fetal heart block and VT in congenital LQTS in the antepartum and postpartum periods.

## Past Medical History

The patient’s history included gestational diabetes, with no previous syncope or seizures. She had 1 spontaneous abortion at age 38 years at 6 weeks’ gestation. The patient has 2 adolescent sons, both full-term deliveries by cesarean section without medical conditions. There is no family history of sudden cardiac death (SCD) or resuscitated sudden cardiac arrest (SCA).

## Differential Diagnosis

Fetal rhythm diagnosis is made by assessment of the relationship of mechanical atrial and ventricular contractions by fetal echocardiography. Fetal magnetocardiography is a modality that offers electrocardiogram (ECG)-like tracings and may aid in the diagnosis of fetal arrythmias.^1^ However, the quality and reliability of the tracings are limited, and this testing is performed at only a few centers.

The differential diagnosis of fetal 2:1 AV conduction includes second-degree heart block, blocked atrial premature beats, and pseudo 2:1 AV conduction caused by extreme prolongation of ventricular action potential in long QT syndrome (LQTS). Second-degree heart block may arise from maternal autoimmune disorders, including Sjogren syndrome, where anti-Ro/anti-La antibody passage to the fetus results in progressive conduction damage. Certain structural heart diseases with L-looped ventricles may also elicit heart block. The differential diagnosis of fetal VT includes intrinsic cardiomyopathy and structural causes such as congenital cardiac hamartomas. Importantly, VT can be associated with congenital LQTS, especially when occurring in conjunction with AV block.^2^

## Investigations

**Figure 2:**
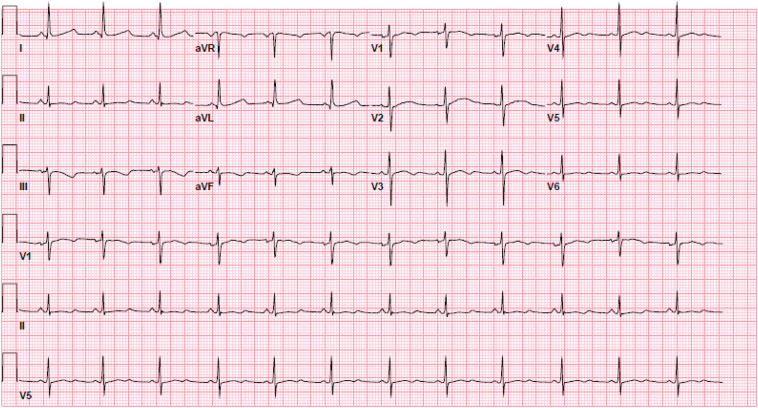
Initial Maternal Electrocardiogram

The mother’s ECG revealed a prolonged QTc of 530 ms (Bazett formula) with a bifid T-wave in lead V_2_ (Figure 2) suggestive of LQTS type II (LQT2). The maternal echocardiogram was unremarkable. Results of evaluation for anti-Ro/anti-La antibodies were negative, and there was no echocardiographic evidence of endocardial fibroelastosis. Genetic evaluation for LQTS revealed a likely pathogenic variant in *KCNH2* (p.Met645Arg) associated with LQT2.

## Management

Before the diagnosis of congenital LQT2 and the exclusion of autoantibody-related AV block, the mother was empirically treated with IVIG and dexamethasone. Although this would be a late presentation anti-Ro/anti-La antibody-mediated AV block, there is a high degree of interest in fetal cardiology to identify and treat autoimmune AV block early, to reverse it or prevent evolution to third-degree AV block.^3^

Following the development of fetal VT, the mother was started on nadolol, 60 mg/d, for maternal and fetal arrhythmia prophylaxis. Intravenous (IV) magnesium was also started at 1 g/h to treat fetal VT. After initiation of these medications, the fetus did not have further VT and returned to predominantly 2:1 conduction on daily fetal echocardiograms. The mother tolerated these therapies without notable side effects; pretreatment magnesium level was 1.7 mg/dL and peaked at 4.3 mg/dL without signs of toxicity. After several days of continuous fetal monitoring and stable fetal echocardiograms without VT, she was transitioned to oral magnesium, which crosses the placenta.

At 35 weeks’ gestation, the fetus was noted to have intrauterine growth restriction and oligohydramnios with preeclampsia, likely related to placental insufficiency. The decision was then made to perform a cesarean delivery.

**Figure 3:**
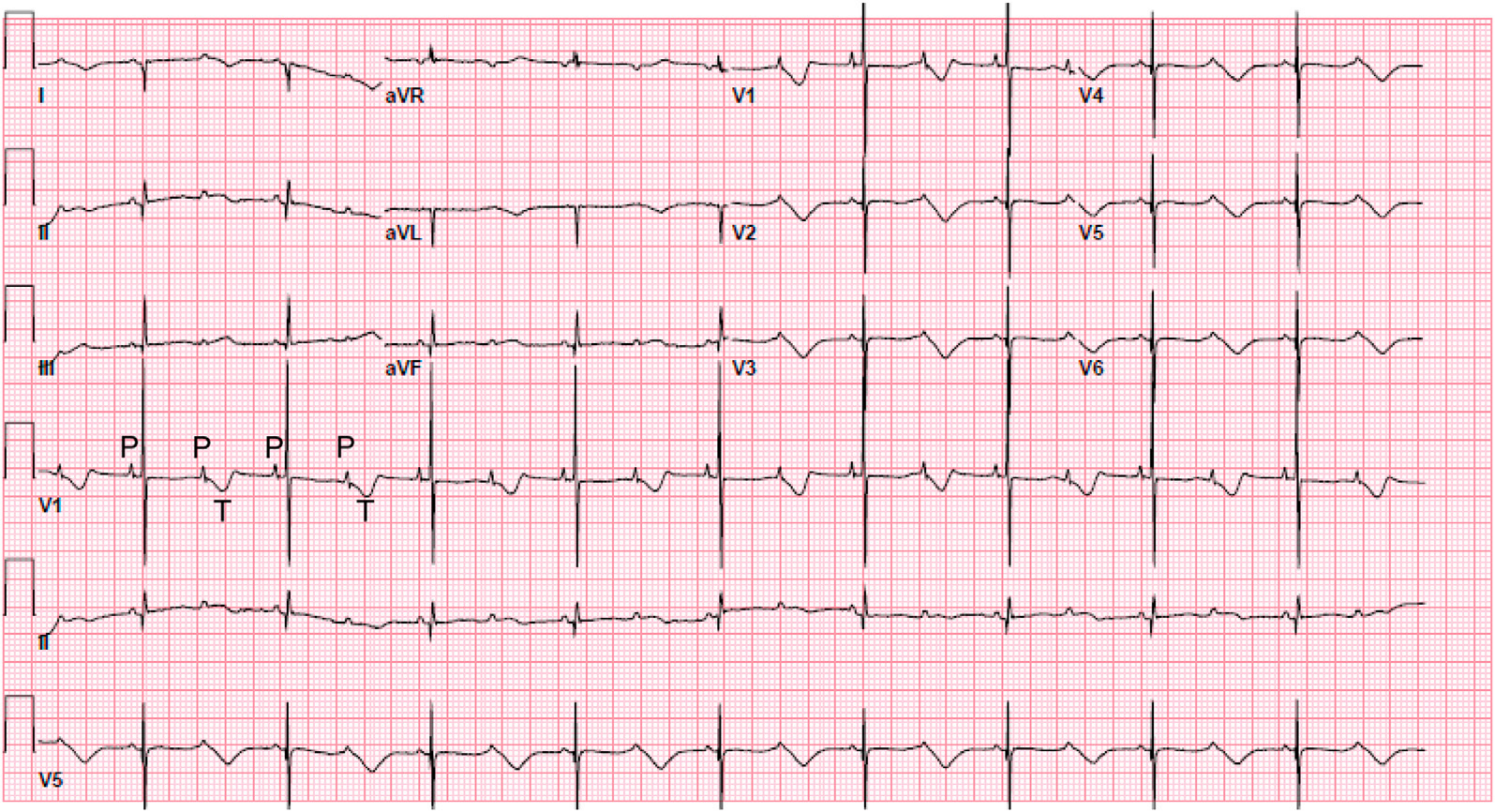
Newborn Electrocardiogram

The first newborn ECG showed a significantly prolonged QTc interval of 794 ms with 2:1 AV block (Figure 3). The infant developed torsades de pointes (TdP) on her first night and was started on propranolol and lidocaine, followed by oral mexiletine at 4 mg/kg every 8 hours. With sodium-channel blockade, the infant’s QTc interval shortened to 474 ms, and she remained in 1:1 AV conduction. Genetic testing of the infant revealed the same variant in *KCNH2* as the mother.

## Discussion

Congenital LQTS represents a group of heritable channelopathies, with 3 major autosomal dominant subtypes (types I, II, and III), that are associated with cardiac repolarization dysfunction and ventricular arrhythmias. LQT2 is associated with pathogenic variants in the *KCNH2* gene causing loss of function in the delayed rectifier channel K_V_11.1 governing the rapid outward current of potassium ions (I_Kr_).^4^ This results in the prolongation of phase 3 of the ventricular action potential and subsequent QT interval prolongation.

Fetal and neonatal 2:1 heart block secondary to LQTS is a result of the ventricle not being repolarized when the next atrial impulse arrives, and treatment that shortens the QT interval may resolve the 2:1 block. Slower ventricular rates with a prolonged QT interval also allow early afterdepolarizations to initiate TdP-type VT. Permanent ventricular pacing at a faster rate, thereby shortening the refractory period, may be useful to shorten the QT interval and prevent TdP.

The risk of serious arrhythmic events (SAEs: SCD or resuscitated SCA) and cardiac events (syncope or SAE) increases with a longer QTc interval, particularly when the QTc interval exceeds 500 ms. Patients with LQT2 have increased mortality between ages 30 and 39 years.^5^ Women with LTQS are at significantly higher risk of SAEs in the postpartum period, as are women with LQT2 who are aged <40 years, regardless of *KCNH2* variant, when compared with men or patients with LQT1.^5^^,^^6^ This risk is hypothesized to result from hormonal modulation of potassium-channel function, specifically because estrogen inhibits I_Kr_ and further prolongs the ventricular action potential.^5^ This patient has a likely pathogenic variant in *KCNH2*, causing a missense mutation that has been observed in patients with LQTS.^7^ This patient represents a higher-risk individual with multiple risk factors for cardiac and SAEs, particularly in the postpartum period.

In the management of LQTS and LQT2, nonselective beta-blockers, preferably nadolol, are first-line therapy. Although mexiletine is particularly effective in LQT3, it also reduces the QT interval in patients with LQT2.^5^ QT-prolonging medications, including flecainide, sotalol, and amiodarone, are avoided. The indications for a primary prevention implantable cardioverter-defibrillator (ICD) are unclear, but this may be considered for patients at higher risk, with syncope, or with a persistent QTc interval ≥500 ms despite pharmacotherapy.^5^^,^^8^

One approach for the management of fetal sustained VT caused by TdP is maternally administered IV magnesium, functioning as a membrane stabilizer that is safely used in pregnancy. Additional agents, including IV lidocaine, beta-blockers, and mexiletine, may also be useful. Beta-blockers cross the placenta to varying degrees, and although transplacental passage is considered undesirable when solely treating the mother, in this instance beta-blockade in the fetus is an intended goal. Neonatal hypoglycemia and bradycardia may be seen in infants exposed to beta-blockers in utero, and there is a small risk of fetal growth restriction with beta-blocker use, although large retrospective studies showed no risk of congenital malformations.^9^

## Follow-Up

For the mother, nadolol was continued, and a primary prevention subcutaneous ICD was placed before discharge, given the presence of multiple risk factors and a QTc interval that remained >500 ms; she continues to do well. The infant continues to receive propranolol (4 mg/kg/d) and mexiletine (12 mg/kg/d) and has remained in normal sinus rhythm on event monitoring after discharge. The infant was prescribed a cardiac monitor, and the parents were trained and provided an automated external defibrillator with child defibrillation patches. Results of ECGs performed on the siblings of the affected infant were normal, and cascade genetic testing is planned. For subsequent pregnancies, there is a 50% chance of inheritance, with variable penetrance and severity in the fetus. As such, in vitro fertilization with preimplant genetic testing would be offered; if this is not pursued, the fetus would be frequently monitored and treated if needed.

## Conclusions

Conduction abnormalities and fetal VT should raise concern for congenital LQTS, especially if clinical features are noted in the mother. In LQTS, adult women are at higher risk for worse outcomes, and this risk increases significantly in the postpartum period.

## Funding Support and Author Disclosures

The authors have reported that they have no relationships relevant to the contents of this paper to disclose.
